# Association Between Infections and Risk of Ankylosing Spondylitis: A Systematic Review and Meta-Analysis

**DOI:** 10.3389/fimmu.2021.768741

**Published:** 2021-10-22

**Authors:** Xiao Zhang, Zhe Sun, Aihong Zhou, Lei Tao, Yingxin Chen, Xinyu Shi, Jia Yin, Zheng Sun, Guoyong Ding

**Affiliations:** ^1^ School of Nursing, Shandong First Medical University & Shandong Academy of Medical Sciences, Taian, China; ^2^ School of Clinical Medline, Shandong First Medical University & Shandong Academy of Medical Sciences, Jinan, China; ^3^ School of Public Health, Shandong First Medical University & Shandong Academy of Medical Sciences, Taian, China; ^4^ The Second Affiliated Hospital of Shandong First Medical University, Taian, China

**Keywords:** ankylosing spondylitis, infections, analytical epidemiology, systematic review, meta-analysis

## Abstract

**Background:**

Previous literature on the association between infections and the risk of developing ankylosing spondylitis (AS) presented controversial results. This meta-analysis aimed to quantitatively investigate the effect of infections on the risk of AS.

**Methods:**

We searched the PubMed, Embase, and Web of Science databases until March 26, 2021 for analytical epidemiological studies on the association between infections and the risk of AS. Fixed or random effect models were used to calculate total risk estimates based on study heterogeneity. Subgroup analysis, and sensitivity analysis were also performed. Publication bias was estimated using funnel plots and Begg’s test.

**Results:**

Six case-control articles (n=1,296,239) and seven cohort articles (n=7,618,524) were incorporated into our meta-analysis. The pooled odds ratio (OR) from these case-control studies showed that infections were associated with an increased risk of AS (OR=1.46, 95% confidence interval [CI], 1.23–1.73), and the pooled relative risk (RR) from the cohort studies showed the same findings (RR=1.35, 95% CI, 1.12–1.63). Subgroup analysis showed that infections in participants with unadjusted comorbidities (OR=1.66, 95% CI, 1.35–2.03), other types of infection (OR=1.40, 95% CI, 1.15–1.70), and infection of the immune system (OR=1.46, 95% CI, 1.42–1.49) were associated with the risk of AS in case-control studies. In cohort studies, infections with adjusted comorbidities (RR=1.39, 95% CI, 1.15–1.68), viral infection (RR=1.43, 95% CI, 1.22–1.66), other types of infection (RR=1.44, 95% CI, 1.12–1.86), and other sites of infection (RR=1.36, 95% CI, 1.11–1.67) were associated with an increased risk of AS.

**Conclusions:**

The findings of this meta-analysis confirm that infections significantly increase the risks of AS. This is helpful in providing an essential basis for the prevention of AS *via* the avoidance of infections.

## Introduction

Ankylosing spondylitis (AS), a complex autoimmune inflammatory rheumatic disease, has long been considered the archetype of spondyloarthritis (SpA). Common symptoms of AS include arthritic symptoms (such as inflammatory back pain, muscle spasms, and sacroiliac arthritis), potential extra-articular symptoms (such as uveitis, psoriasis, and inflammatory bowel syndrome), and the involvement of the heart, bone, lung, kidneys, and skin (1, 2). The worldwide prevalence of AS ranges between 0.07% and 0.32% (3). In addition, clinical symptoms of patients with AS usually appear between the ages of 26 and 45 years. Men also are more likely to suffer from AS than women, the prevalence being two to three times higher in men than in women (4–6).

The pathogenesis of AS is complex and multifactorial. Early studies have confirmed that AS is strongly associated with the inheritance of HLA allele B27, which might misfold in the endoplasmic reticulum (ER), leading to the upregulation of interleukin (IL)-23 in dendritic cells (7–9). It may also result in the presentation of intracellular peptides to T cells, which may trigger cross reactions, leading to tissue inflammation (10, 11). Several recent studies have emphasized the critical role of intestinal flora dysregulation in the development and progression of AS, and have suggested that 60% of AS patients are associated with subclinical intestinal inflammation (12–14). This might be related to the imbalance of IL-17 or IL-23 cytokines caused by the activation a Th17-mediated immune response by intestinal dysbiosis (15). In contrast, the role of environmental factors in the etiology of AS is far from clear. One of the most popular theories presume that the onset of AS in susceptible individuals may be caused by infections (16), and that infections have the potential to modulate and attenuate immune responses.

The underlying pathogenic mechanisms for linking infections and AS involve changes in target cells and immune cells, and antigenic cross-reactions between microbial and host determinants (17). Infections might cause the quantitative reduction in specific T cells and the host defense defect against the infections that allows microbial antigens to reach the joint (18). The association between the infections and AS may be *via* IL-17 or C reactive protein levels that can induce inflammatory response (10, 19). In addition, certain microbial infections may reduce CD4+ T cells, and protein fragments released by dying CD4 lymphocytes may induce autoreactive CD8 lymphocytes (20). There is evidence of significantly elevated levels of IL-6 and TNF-a in AS patients, which might be caused by infections (21).

Numerous studies have investigated AS-related infections, including bacterial (10, 17, 18, 22), viral (17, 19, 20, 23), fungal (11), and those by microorganisms with sizes between those of bacteria and viruses (18, 24, 25). The infected sites include the respiratory (18, 24–26), immune (20, 23, 26, 27), digestive (10, 22, 26, 28), and genitourinary systems (19). However, there is no consensus on the association between infections and the risk of AS. To our knowledge, no systematic review and meta-analysis to date has investigated the effect of infections on the risk of AS. Therefore, in order to obtain a more convincing conclusion, this study aimed to review all the relevant studies and summarise the findings, in order to investigate the association between infections and AS.

## Materials and Methods

The current study was developed according to the guidelines for the Meta-Analysis of Observational Studies in Epidemiology (MOOSE) (29) and Preferred Reporting Items for Systematic Review and Meta-Analyses (PRISMA, **Supplementary Table 1**) (30). The protocol is presented in **Supplementary Data**.

### Search Strategy

We systematically searched the PubMed, Embase, and Web of Science electronic databases to identify such literature published up until March 26, 2021, using terms related to infection and AS. The search strategy was developed and implemented under the guidance of experts on library services from Shandong First Medical University. The main search strategy involved the following: (spondylitis, ankylosing OR spondyloarthritis ankylopoietica OR ankylosing spondylarthritis OR ankylosing spondylarthritides OR spondylarthritides, ankylosing) AND (infections OR enteritis OR salmonella OR pneumonia OR klebsiella pneumoniae OR urogenital infections OR paradentitis OR tonsillitis OR infection of the upper respiratory tract OR appendicitis OR gastritis OR helicobactor pylori OR virus) AND (case-control study OR retrospective study OR cohort study OR prospective study OR longitudinal study OR follow-up study). The complete search strategy of the three databases is listed in **Supplementary Table 2**. Moreover, only English- or Chinese-language literature was retrieved from the databases as the investigators were proficient in both these languages. The lists of references from all of the included studies were manually checked to identify possible additional studies.

### Selection Criteria

Studies were included according to the following criteria: (1) the study design was a cohort or case-control study; (2) the studies defined infections using self-reporting, clinical diagnosis, or basic medical experiment, and focused on infections that developed before AS did; (3) the outcome of interest was AS; and (4) the studies provided the effect size (relative risk [RR], hazard ratio [HR], or odds ratio [OR] with 95% confidence interval [CI]) or raw data that could be used to calculate RR, HR, or OR. The exclusion criteria were as follows: (1) non-human-based studies; (2) studies that were poster presentations, reviews, conference summaries, or dissertations; and (3) the scores of quality evaluation according to the Newcastle-Ottawa Scale (NOS) were <4 (31). In the situation of multiple eligible studies from the same population, only the study with the largest number of individuals was included. Two authors (X.Z. and A.Z.) independently screened titles and abstracts initially and then evaluated full-text articles to ensure the included studies met the eligible inclusion criteria. Any disagreement between them was settled by another author (G.D.).

### Data Extraction and Quality Assessment

According to the study design, the included studies were divided into two extraction forms of case-control studies and cohort studies. The following data were extracted from the eligible case-control studies using a customized form: the first name of the first author, year of publication, study location, types of infection, definition of infection, definition of AS, age, sex, sample size, adjustment for potential confounding factors, and estimates of association. The follow-up duration in cohort studies was also included. The Cochrane Non-randomized Studies Methods Working Group recommended the use of the NOS to assess the quality of observational studies (range: 0–9 stars) (32). According to the score stars of the NOS, the included studies were defined as low- (1–3 stars), moderate- (4–6 stars), and high-quality (7–9 stars). Therefore, if the study obtained ≥4 stars, it was considered to have an above-moderate quality and, thus, was incorporated into our meta-analysis (31). Data extraction and quality assessment were conducted by two independent investigators (L.T. and Y.C.), and disagreements between them were resolved through negotiation with a third researcher (Z.S.).

### Statistical Analysis

The statistical analyses were performed using Stata 15.1 software (Stata Corp, College Station, TX, USA). All of the tests were bilateral, and *P* values <0.05 were considered statistically significant, though *P* values >0.1 illustrated no heterogeneity among studies in the heterogeneity test (33). ORs, RRs, or HRs and their corresponding 95% CIs were considered to be the effect values of different infections on the risk of AS. The pooled OR and RR with their corresponding 95% CIs were used in case-control and cohort studies, respectively, to assess the association of infection with the risk of AS. We used the *Q* test and the *I* ^2^ statistic to detect heterogeneity among the studies. *I* ^2^ describes the percentage of total variation due to heterogeneity among studies rather than due to chance (34). In the presence of high heterogeneity (*I* ^2^>50%), the Dersimonian and Laird random effects model (REM) was adopted as the pooling method; otherwise, the Mantel-Haensze fixed effects model (FEM) was applied as the pooling method.

Subgroup analyses were performed based on adjusting for comorbidities, infection type, and infection site. In addition, considering that publication year, study location, sample size, definition of infection, and duration of follow-up (only in cohort studies) may affect between-study heterogeneity, subgroup analysis was also conducted based on these possible factors. Sensitivity analyses were performed to validate the stability of pooled ORs of case-control literature and pooled RRs of cohort literature by removing each individual study. In addition, we used the funnel plot and Begg’s test to assess publication bias.

## Results

### Literature Search and Study Selection

**Figure 1:**
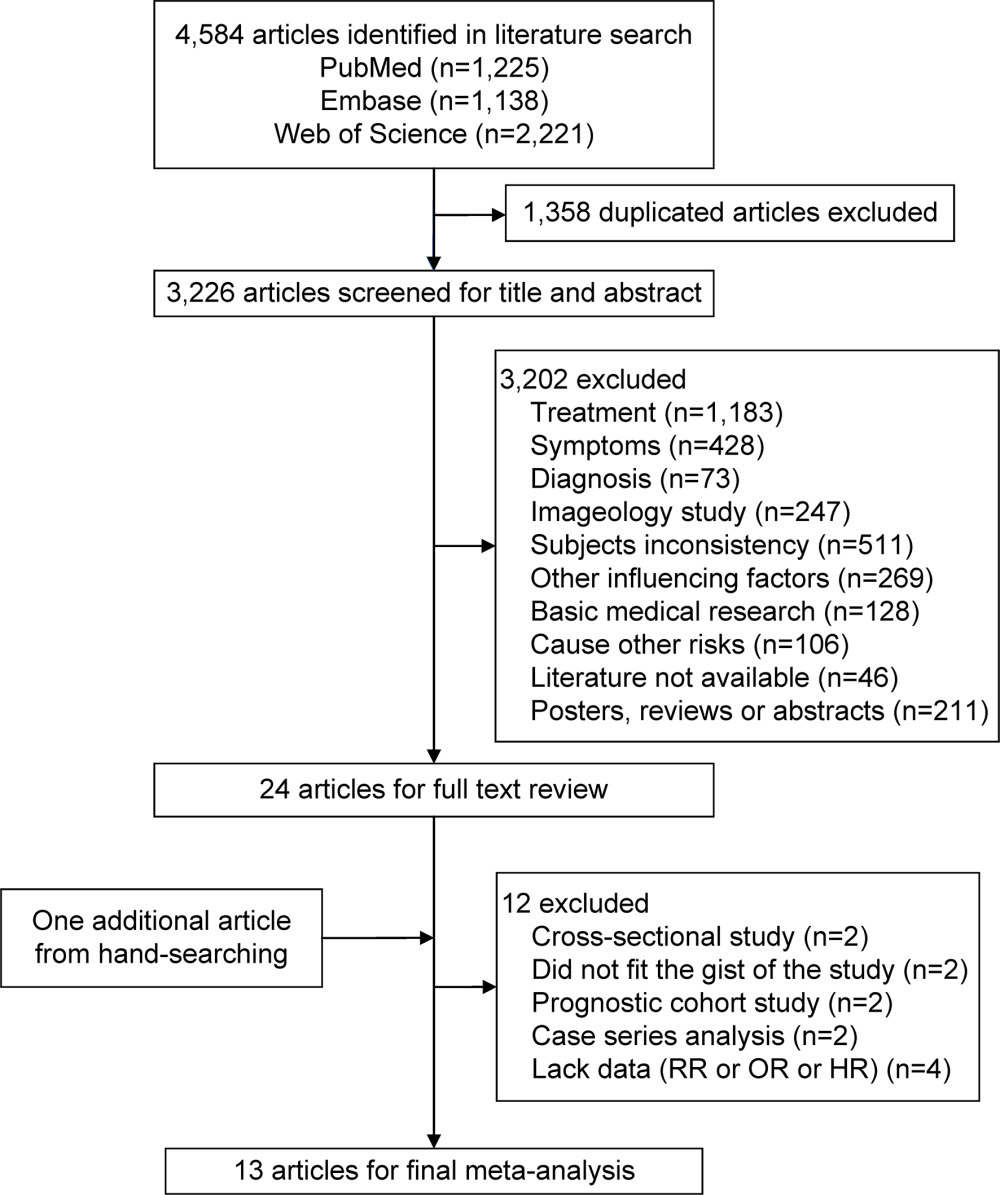
Flowchart of the selection of studies included in this meta-analysis. HR, hazard ratio; OR, odds ratio; RR, relative risk.

The flowchart of the literature search and study selection process is represented in **Figure 1**. Using three electronic databases and running the search strategy, a total of 4,584 potentially relevant articles were identified. In total, 1,358 duplicate articles were excluded. An additional 3,226 articles were excluded by screening for the title and abstract, leaving 24 articles for the full-text review. Screening *via* hand-searching found 1 relevant article. An additional 12 articles were excluded because they did not meet the inclusion criteria. Therefore, 13 articles that met the inclusion criteria were ultimately included (10, 11, 17–20, 22–28).

### Study Characteristics

There were six case-control design articles (18, 22, 25–28) and seven cohort study design articles (10, 11, 17, 19, 20, 23, 24). It must be noted that one article involved two case-control studies (28). Therefore, we included 13 articles with 14 studies. The characteristics of the included studies, which were published from 2004 to 2020, are summarized in **Supplementary Tables 3**–**6**. The case-control studies included 1,296,239 participants; the cohort studies, 7,618,524 participants.

Seven studies were from Asia (11, 19, 22–25, 27), five, from Europe (10, 17, 26, 28), and two, from North America (18, 20). The mean NOS star score of the methodological quality of the studies was 7.9. Two articles (with three studies) obtained 5 stars (25, 28); two studies, 7 stars (17, 23); two studies, 8 stars (10, 26); and seven studies, 9 stars (11, 18–20, 22, 24, 27). The follow-up duration ranged from 3.6 to 34 years in the cohort studies. All studies involved both men and women (10, 11, 18–20, 22–28) except the study by Nielsen et al. (17). Nine studies defined the infection using clinical diagnosis (11, 17, 19, 20, 22–24, 26, 27), two studies (in the same article) defined the infection using self-report (28), and three studies defined the infection using laboratory tests in etiologic diagnoses (10, 18, 25). Thirteen studies reported one type of infection (10, 11, 19, 20, 22–28), and one study reported four types of infections (18). In the studies, except for those by Feng et al. and Yen et al., the risk estimates were adjusted for confounding variables, such as age, sex, urbanization, income, comorbidities, body mass index, educational attainment, smoking status, and alcohol consumption (10, 11, 17–20, 22, 24, 26–28).

### Infection and the Risk of AS

**Figure 2:**
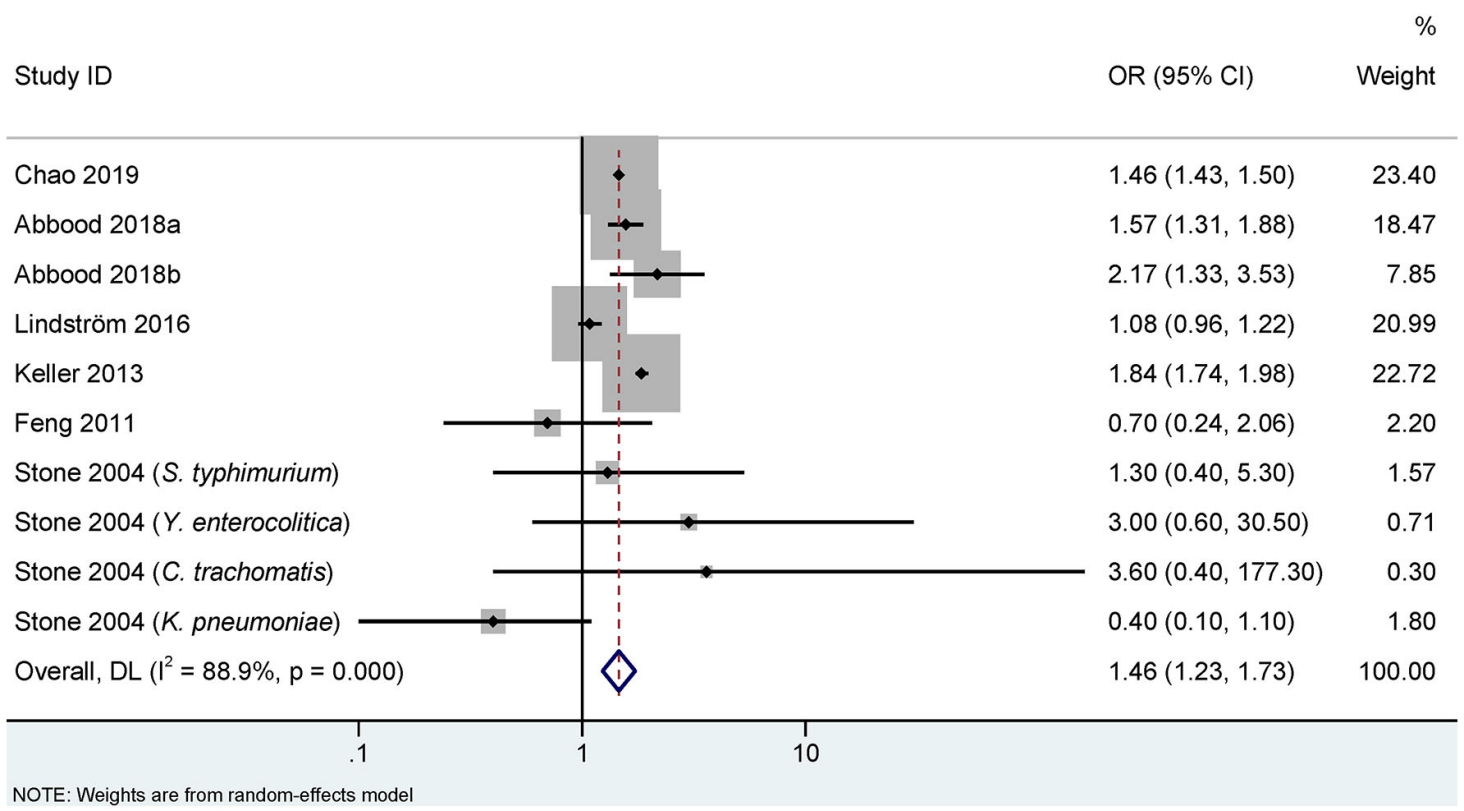
Random effects meta-analysis of the association between infections and ankylosing spondylitis in case-control studies. CI, confidence interval; OR, odds ratio. a represents case-control study of self-reported ankylosing spondylitis; b represents case-control study of clinical recorded ankylosing spondylitis.

**Figure 3:**
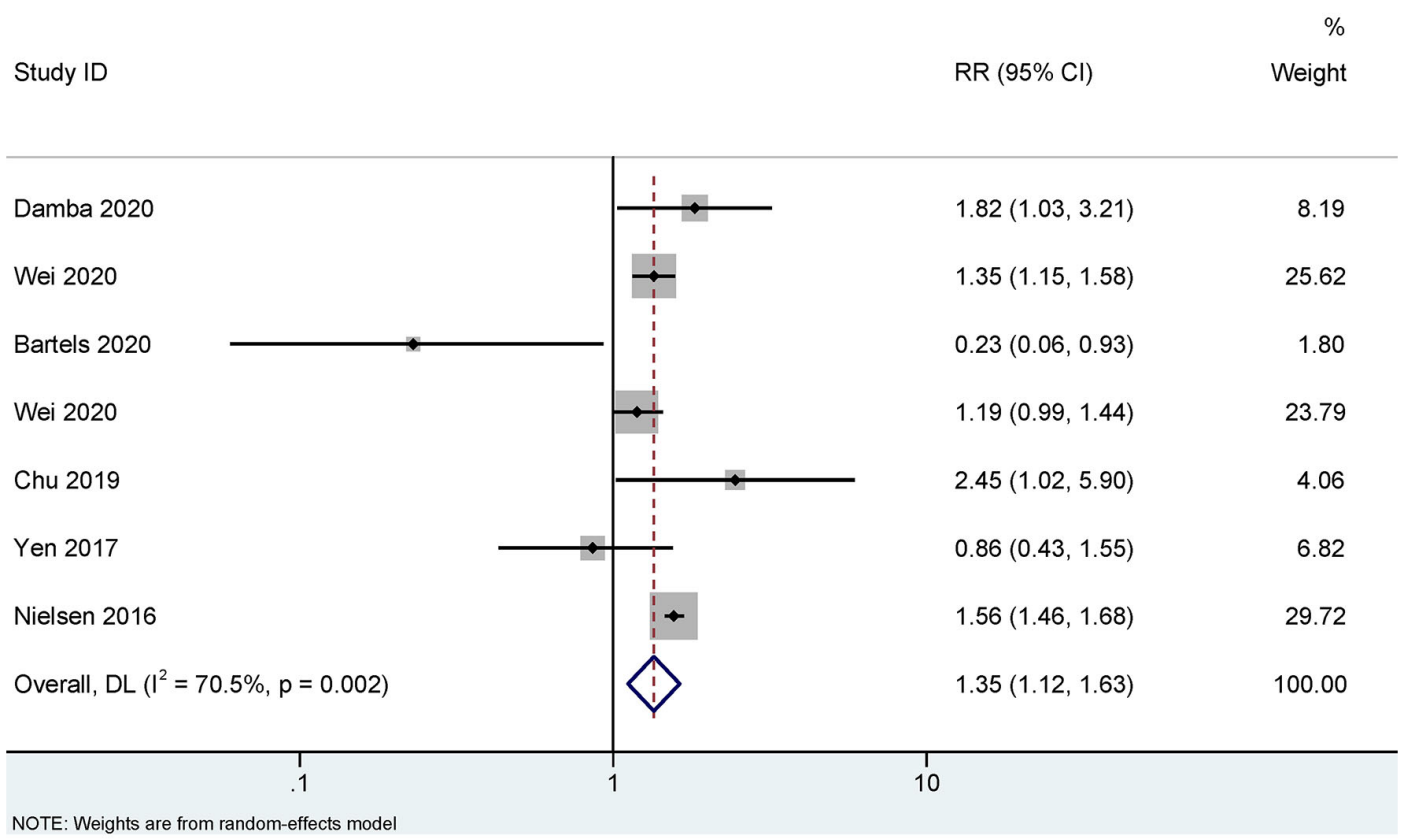
Random effects meta-analysis of the association between infections and ankylosing spondylitis in cohort studies. CI, confidence interval; RR, relative risk.

**Figures 2**, **3** show the association between infections and the risk of AS in seven case-control studies (18, 22, 25–28) and seven cohort studies (10, 11, 17, 19, 20, 23, 24), respectively. There were significant heterogeneities among the seven case-control studies (*P_Q_* <0.001. *I* ^2 =^ 88.9%), and the seven cohort studies (*P_Q_* <0.001. *I* ^2 =^ 70.5%). Therefore, REM was used to pool the OR for case-control studies and RR for cohort studies. Our meta-analysis showed that, compared to the control group, the infection group was significantly associated with an increased risk of AS. The pooled OR calculated by REM was 1.46 (95% CI, 1.23–1.73) for the case-control studies, and the pooled RR was 1.35 (95% CI, 1.12–1.63) for the cohort studies.

### Subgroup Analysis

**Table 1** shows the subgroup analysis based on comorbidities (adjusted *vs*. unadjusted), infection type (bacterial *vs*. viral *vs*. other), and infection site (immune system *vs*. other).

**Table 1:** The subgroup analyses of the association between infections and the risk of AS.

Subgroups	No. of items	OR/RR/HR (95% CI)	*I* ^2^ (%)	Chi-square test *P*-value
**Case-control studies**				
**Comorbidities**				0.139
Adjusted	2	1.26 (0.94–1.70)	95.7	
Unadjusted	8	1.66 (1.35–2.03)	45.8	
**Infection Type**				0.864
Bacterial	4	1.31 (0.61–2.78)	55.3	
Other^*^	6	1.40 (1.15–1.70)	82.7	
**Infection Site**				0.347
Immune system	2	1.46 (1.42–1.49)	0.0	
Other^**^	10	1.27 (0.96–1.69)	86.7	
**Cohort studies**				
**Comorbidities**				0.157
Adjusted	6	1.39 (1.15–1.68)	71.6	
Unadjusted	1	0.86 (0.45–1.63)	0.0	
**Infection Type**				0.766
Bacterial	2	0.70 (0.10–4.78)	87.4	
Viral	4	1.43 (1.22–1.66)	35.3	
Other^*^	3	1.44 (1.12–1.86)	74.0	
**Infection Site**				0.863
Immune system	2	1.27 (0.61–2.65)	66.0	
Other^**^	5	1.36 (1.11–1.67)	76.6	

AS, ankylosing spondylitis; CI, confidence interval; HR, hazard ratio; OR, odds ratio, RR, relative risk.

^*^Other types of infection include fungi, chlamydia, and mycoplasma.

^**^Other sites of infection include the digestive system, respiratory system, and genitourinary system.

In the case-control studies, our analysis showed no significant difference between adjusted comorbidities (OR=1.26, 95% CI, 0.94–1.70) and unadjusted comorbidities (OR=1.66, 95% CI, 1.35–2.03) (*P*=0.139). Similar results were reported with the infection type (bacterial, OR=1.31, 95% CI, 0.61–2.78; other, OR=1.40, 95% CI, 1.15–1.70; *P*=0.864) and the infection site (immune system, OR=1.46, 95% CI, 1.42–1.49; other, OR=1.27, 95% CI, 0.96–1.69; *P*=0.347).

In the cohort studies, our analysis showed no statistically significant difference between adjusted comorbidities (RR=1.39, 95% CI, 1.15–1.68) and unadjusted comorbidities (RR=0.86, 95% CI, 0.45–1.63; *P*=0.157). No differences were also found in the infection type (bacterial, RR=0.70, 95% CI, 0.10–4.78; viral, RR=1.43, 95% CI, 1.22–1.66; other, RR=1.44, 95% CI, 1.12–1.86; *P*=0.766) and infection site (immune system, RR=1.27, 95% CI, 0.61–2.65; other, RR=1.36, 95% CI, 1.11–1.67; *P*=0.863).

We also conducted subgroup analysis based on publication year, study location, sample size, definition of infection, and duration of follow-up. As shown in **Supplementary Table 7**, the results showed no significant difference between those subgroups in the case-control studies, which indicated that these above factors were not the source of heterogeneity in case-control studies. In the cohort studies, only for definition of infection, there was a significant deference between clinical diagnosis (RR=1.39, 95% CI, 1.19–1.63, *I* ^2 ^= 62.3%) and basic medical experiment (RR=0.23, 95% CI, 0.06–0.91, *I* ^2 ^= 0%, *P*=0.010). Definition of infection might be one of the sources of heterogeneity in the cohort studies.

### Sensitivity Analysis and Publication Bias Detection

**Figure 4:**
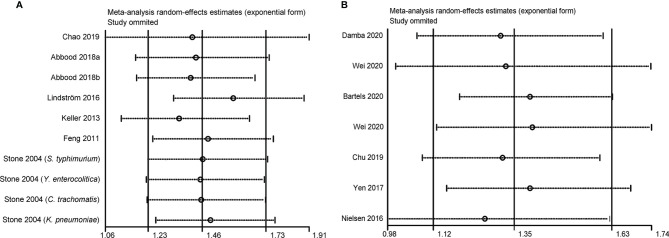
Sensitivity analysis of the association between infections and ankylosing spondylitis. **(A)** Case-control study; a represents case-control study of self-reported ankylosing spondylitis; b represents case-control study of clinical recorded ankylosing spondylitis; **(B)** Cohort study.

As shown in **Figure 4**, the results of sensitivity analysis showed that no individual study significantly influenced the pooled OR in the case-control studies. However, the study by Nielsen et al. affected the pooled RR in the cohort studies. The pooled RR was 1.26 (95% CI, 0.98–1.62; *I* ^2 ^= 58.6%) by omitting this study.

**Figure 5:**
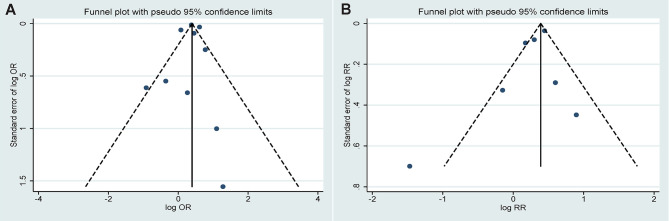
Funnel plots corresponding to the random effects meta-analysis of the association between infections and ankylosing spondylitis. **(A)** Case-control study (*P*=0.721 by Begg’s test); **(B)** Cohort study (*P*=0.368 by Begg’s test).

The funnel plots for estimating publication bias were roughly symmetrical for the case-control (**Figure 5A**) and cohort studies (**Figure 5B**). No publication bias was detected by Begg’s test for the case-control (*P*=0.721) and cohort studies (*P*=0.368).

## Discussion

To the best of our knowledge, this meta-analysis is the first study to investigate the association between infections and the risk of developing AS. The current meta-analysis included seven case-control studies with 1,296,239 participants and seven cohort studies with 7,618,524 participants. The results of this study confirm that infections play an important role in the risk of AS. Determining the effect of infections on AS would be beneficial for the identification of those who are at higher risk of AS as reasonable preventive intervention can be conducted for this population, enabling a far-reaching significance for the prevention of AS.

The results showed that infections are associated with an increased risk of AS in both the case-control and cohort studies. Our findings are consistent with most of the studies, including the four studies from Asia (19, 22, 24, 27), three studies from Europe (17, 28), and one study from North America (20). However, the study by Bartels et al. showed opposite results (10), in that previous *Helicobacter pylori* (*H.pylori*) infection may reduce the risk for developing AS. Another study found that *H. pylori* infection was eradicated in more than 80% of cases in the same cohort as that of Bartels et al. (35), indicating that *H. pylori* leaves a protective potential for the development of AS later in life when it is eradicated (10). Furthermore, the microbiota in the gastrointestinal tract changes after the eradication of *H. pylori*, which may have an impact on AS development (36, 37).

Our subgroup analyses showed that there was an association between infection and the risk of AS after adjusting for comorbidities in the cohort studies, which suggested that the comorbidities are a remarkably important confounding factor in cohort studies, and that we must control and adjust it. However, an association between infection and the risk of AS was found in the case-control studies without adjusted comorbidities. This is due to the nature of the case-control study design. As one of the matching factors of case-control studies, comorbidities may be matched in the design stage, cancelling the need to adjust for comorbidities in the statistical analysis stage.

With regards to the infection types, we did not observe that bacterial infections contribute to the risk of AS. In case-control studies, only the study by Keller et al. showed that there is an association between AS and a prior diagnosis of chronic periodontitis, which is characterized by an oral bacterial infection (22). This may be because rheumatic diseases and chronic periodontitis share pathogenic factors, including a dysfunction of inflammatory mechanisms and an imbalance of proinflammatory and anti-inflammatory cytokines (22, 38–40). In the cohort studies, the study by Bartels et al. showed that *H. pylori* may be a protective factor for AS (10). The study of Nielsen et al. also showed that bacterial infection is associated with the development of AS in the general population (17). More cohort studies are needed to verify whether bacterial infection causes AS. The result of the subgroup of other infection types was that other infection types are associated with AS in both the case-control and cohort studies. In our analysis, other types of infection included fungal, chlamydia, and mycoplasma. The pathogenesis of AS due to other types of infection is far from clear. For example, one study suggested that AS could be induced after exposure to *Candida albicans* through a T cell-driven model towards Th17 responses (11). Another study suggested that *Mycoplasma pneumoniae* has a significant impact on immune cells and the immune system of the host, including polyclonal activation of T and B cells and the secretion of related cytokines (24), leading to a breakdown of immune-tolerance. In addition, subgroup analysis indicated that viruses play an important role in the risk of AS in the cohort studies. One study suggests that viruses (e.g., human papillomavirus) might lead to inflammatory or immune-mediated disease by activating the pathogenic IL-23/IL-17 axis, resulting in elevated serum levels of Th17 cells, IL-17, and IL-23, and the imbalance of IL-17A/IL-23 cytokines (19). In the subgroup analysis of infection sites, we found that the infection of the immune system was significantly associated with the risk of AS in the case-control studies. Some immune organs, such as the tonsils, are involved in allergens tolerance by generating allergen-specific FOXP3^+^ regulatory T cells, suggesting that they are critical in the development of immune-tolerance (41). Some studies postulated that the alteration of immune tolerance in patients with tonsillitis might lead to the inflammatory disorders in autoimmune arthritis, including AS; therefore, tonsillitis might be aggravated by spondylitis, leading to the diagnosis of AS (26, 27). In addition, the higher risks of AS among infected people might be explained by HIV-induced antigen-driven immune responses (42), T cell imbalance (43), and molecular mimicry located between HIV protein and self-antigens (44). For the cohort studies, the infections in other sites were significantly associated with the risk of AS, which indicates that AS might be triggered by respiratory tract infections and genitourinary system infections. The pathogenesis of AS caused by the infection in the genitourinary system is mixed. In one of them, human papillomavirus in the genitourinary system might lead to AS by activating the IL-23/IL-17 axis (19). For the respiratory system, *Klebsiella pneumoniae* might lead to a decrease in the number of specific T cells, which could reflect an insufficient in the host’s defense against *Klebsiella*, thereby allowing AS to be affected by bacterial antigens that reach the joint (18). In our study, we found that some design types were meaningful and some were not for the same subgroups, which might be related to the small number of included articles or the large heterogeneity between the included studies.

This meta-analysis has the following strengths: pooled effect values were analysed according to the different study design, and we grouped the studies according to the types and sites of infection to determine whether these factors were associated with the risk of AS. This study included analytical epidemiological studies to determine the risk of AS, and the sample size was large. The included studies were adjusted for potential confounding variables, which improved the accuracy of risk estimates.

However, some limitations have affected the current study. First, although heterogeneity was explored *via* subgroup analysis, it was still very high, which may be related to age, sex distribution of participants, definition of infection, diagnosis of AS, etc. The subgroup analysis suggested that definition of infection was one of sources of heterogeneity in the cohort studies, which indicated that the possible disagreement between measurement methods might be a source of misclassification. But heterogeneity within subgroups remained high. In addition, although we extracted the definition of AS, only two articles declared that the diagnosis of AS was based on the Amor criteria (18, 25). As most of articles were retrospective, the International Classification of Diseases codes for the diagnoses of AS were based on records made by physicians and hospitals rather than a prospective clinical setting; thus, we could not set uniform criteria for the definition of AS, which also may result in heterogeneity. Second, this meta-analysis included only English- and Chinese-language articles, and qualified articles in other languages were not included in the analysis, which might have affected the pooled estimated value. Third, our pooled effect is affected by the study by Nielsen et al. in the cohort studies. However, the study by Nielsen et al. has the largest weight when synthesizing RRs across studies, because of its large sample size, narrow confidence interval, and high quality. When the study by Nielsen et al. was omitted in the sensitivity analysis, the pooled effect estimate was affected by some low-quality studies due to the increased weights. Thus, more large cohort studies are recommended in the future to assess the impact of infections on the risk of AS.

In conclusion, this meta-analysis confirms that there is an association between infections and an increased risk of AS, although the included studies suffered from high heterogeneity. As much as the mechanism of infection and the effect of bacterial and viral infections on AS has not yet been determined, further studies, particularly more higher quality prospective cohort studies and case-control studies, are required to verify that there is a true cause-and-effect relationship between infections and the risk of developing AS.

## Data Availability Statement

The original contributions presented in the study are included in the article/**Supplementary Material**. Further inquiries can be directed to the corresponding author.

## Author Contributions

GD and XZ designed the study protocol. XZ, XS, and JY conducted the literature search. XZ, AZ, and GD retrieved and selected the articles. LT, YC, and ZhengS conducted data extraction. XZ and ZheS performed the statistical analysis of the data. XZ, ZheS, and GD wrote the manuscript draft. GD and ZhengS supervised the study. All authors contributed to the article and approved the submitted version.

## Funding

This work was supported by the Natural Science Foundation of Shandong Province for the General Program (Grant No. ZR2020MH339). The funder had no role in study design, data collection and analysis, decision to publish, or preparation of the manuscript.

## Conflict of Interest

The authors declare that the research was conducted in the absence of any commercial or financial relationships that could be construed as a potential conflict of interest.

## Publisher’s Note

All claims expressed in this article are solely those of the authors and do not necessarily represent those of their affiliated organizations, or those of the publisher, the editors and the reviewers. Any product that may be evaluated in this article, or claim that may be made by its manufacturer, is not guaranteed or endorsed by the publisher.
