# Supplementary material for: Association Between Infections and Risk of Ankylosing Spondylitis: A Systematic Review and Meta-Analysis
Source: Front Immunol. 2021 Oct 22;12:768741. doi: 10.3389/fimmu.2021.768741 (PMC8569302; doi:10.3389/fimmu.2021.768741)
Supplement: Supplementary file 1 [file DataSheet_1.doc]

**Supplementary Material**

**Contents**

[Supplementary Table 1. PRISMA checklist. 1](#__RefHeading___Toc84112781)

[Supplementary Data. Protocol. 3](#__RefHeading___Toc84112782)

[Supplementary Table 2. Search strategy. 6](#__RefHeading___Toc84112783)

[Supplementary Table 3. Characteristics of included case-control studies on infections and AS. 9](#__RefHeading___Toc84112784)

[Supplementary Table 4. Other characteristics of included case-control studies on infections and AS. 10](#__RefHeading___Toc84112785)

[Supplementary Table 5. Characteristics of included cohort studies on infections and AS. 12](#__RefHeading___Toc84112786)

[Supplementary Table 6. Other characteristics of included cohort studies on infections and AS. 13](#__RefHeading___Toc84112787)

[Supplementary Table 7. The subgroup analyses of the association between infections and the risk of AS based on possible factors.. 14](#__RefHeading___Toc84112788)

# Supplementary Table 1. PRISMA checklist.

| **Section/topic** | **#** | **Checklist item** | **Reported on page #** |
| --- | --- | --- | --- |
| **TITLE** |  |  |  |
| Title | 1 | Identify the report as a systematic review, meta-analysis, or both. | 1 |
| **ABSTRACT** |  |  |  |
| Structured summary | 2 | Provide a structured summary including, as applicable: background; objectives; data sources; study eligibility criteria, participants, and interventions; study appraisal and synthesis methods; results; limitations; conclusions and implications of key findings; systematic review registration number. | 1 |
| **INTRODUCTION** |  |  |  |
| Rationale | 3 | Describe the rationale for the review in the context of what is already known. | 2 |
| Objectives | 4 | Provide an explicit statement of questions being addressed with reference to participants, interventions, comparisons, outcomes, and study design (PICOS). | 2 |
| **METHODS** |  |  |  |
| Protocol and registration | 5 | Indicate if a review protocol exists, if and where it can be accessed (e.g., Web address), and, if available, provide registration information including registration number. | 2 |
| Eligibility criteria | 6 | Specify study characteristics (e.g., PICOS, length of follow-up) and report characteristics (e.g., years considered, language, publication status) used as criteria for eligibility, giving rationale. | 2 |
| Information sources | 7 | Describe all information sources (e.g., databases with dates of coverage, contact with study authors to identify additional studies) in the search and date last searched. | 2 |
| Search | 8 | Present full electronic search strategy for at least one database, including any limits used, such that it could be repeated. | 2 |
| Study selection | 9 | State the process for selecting studies (i.e., screening, eligibility, included in systematic review, and, if applicable, included in the meta-analysis). | 2 |
| Data collection process | 10 | Describe method of data extraction from reports (e.g., piloted forms, independently, in duplicate) and any processes for obtaining and confirming data from investigators. | 3 |
| Data items | 11 | List and define all variables for which data were sought (e.g., PICOS, funding sources) and any assumptions and simplifications made. | 3 |
| Risk of bias in individual studies | 12 | Describe methods used for assessing risk of bias of individual studies (including specification of whether this was done at the study or outcome level), and how this information is to be used in any data synthesis. | NA |
| Summary measures | 13 | State the principal summary measures (e.g., risk ratio, difference in means). | 3 |
| Synthesis of results | 14 | Describe the methods of handling data and combining results of studies, if done, including measures of consistency (e.g., I2) for each meta-analysis. | 3 |
| Risk of bias across studies | 15 | Specify any assessment of risk of bias that may affect the cumulative evidence (e.g., publication bias, selective reporting within studies). | 3 |
| Additional analyses | 16 | Describe methods of additional analyses (e.g., sensitivity or subgroup analyses, meta-regression), if done, indicating which were pre-specified. | 3 |
| **RESULTS** |  |  |  |
| Study selection | 17 | Give numbers of studies screened, assessed for eligibility, and included in the review, with reasons for exclusions at each stage, ideally with a flow diagram. | 3 |
| Study characteristics | 18 | For each study, present characteristics for which data were extracted (e.g., study size, PICOS, follow-up period) and provide the citations. | 3 |
| Risk of bias within studies | 19 | Present data on risk of bias of each study and, if available, any outcome level assessment (see item 12). | NA |
| Results of individual studies | 20 | For all outcomes considered (benefits or harms), present, for each study: (a) simple summary data for each intervention group (b) effect estimates and confidence intervals, ideally with a forest plot. | 3 |
| Synthesis of results | 21 | Present results of each meta-analysis done, including confidence intervals and measures of consistency. | 3 |
| Risk of bias across studies | 22 | Present results of any assessment of risk of bias across studies (see Item 15). | 7 |
| Additional analysis | 23 | Give results of additional analyses, if done (e.g., sensitivity or subgroup analyses, meta-regression [see Item 16]). | 4,6,7 |
| **DISCUSSION** |  |  |  |
| Summary of evidence | 24 | Summarize the main findings including the strength of evidence for each main outcome; consider their relevance to key groups (e.g., healthcare providers, users, and policy makers). | 7-8 |
| Limitations | 25 | Discuss limitations at study and outcome level (e.g., risk of bias), and at review-level (e.g., incomplete retrieval of identified research, reporting bias). | 8 |
| Conclusions | 26 | Provide a general interpretation of the results in the context of other evidence, and implications for future research. | 8 |
| **FUNDING** |  |  |  |
| Funding | 27 | Describe sources of funding for the systematic review and other support (e.g., supply of data); role of funders for the systematic review. | 8 |

# Supplementary Data. Protocol.

**Association between infections and risk of ankylosing spondylitis: A systematic review and meta-analysis**

**Background**

Ankylosing spondylitis (AS), a complex autoimmune inflammatory rheumatic disease, has long been considered the archetype of spondyloarthritis (SpA). AS has typical radiographic manifestations, with the common symptoms of AS including arthritic symptoms (such as inflammatory back pain, muscle spasms, sacroiliac arthritis), potential extra-articular symptoms (such as uveitis, psoriasis, inflammatory bowel syndrome), as well as the involvement of the heart, bone, lung, kidney and skin^1,2^. The worldwide prevalence of AS ranges between 0.07% and 0.32%^3^.

The pathogenesis of AS is quite complex and multifactorial. Early studies have confirmed that AS is associated with the inheritance of HLA allele B27^4-6^. And another study found that the etiology of AS is related to the imbalance of IL-17A /IL-23 cytokines^7^. Recently, one of the most popular theories presume that the onset of AS in susceptible individuals may be caused by infections^8^, and that infections have the potential to modulate and attenuate immune responses. It has been suggested that the underlying pathogenic mechanisms for linking infection and AS involve changes in target cells, changes in immune cells, immune responses response to the determinant shared by host and virus, and cross-reactions between idiotype and antiviral antibodies and their respective autoantigens^9^.

**Aim/Objectives**

This study will aim to determine all relevant studies and summary the findings to quantitatively investigate the association between infection and AS.

**Methods**

**Eligibility criteria**

Participants/Population: Participants with AS.

Intervention(s), exposure(s): Patients with infections (defined using self-reporting, clinical diagnosis, or basic medical experiment) that developed before ankylosing spondylitis.

Outcomes: Interest is outcome data on association between infection and the risk of AS.

Measures of effect: Relative risk (RR), hazard ratio (HR), or odds ratio (OR) with 95% confidence interval (CI) or raw data that could be used to calculate RR, HR, or OR.

Study designs: Case-control or cohort studies.

Language: English or Chinese languages restriction.

**Information sources/search strategy**

A systematic search will be performed throughout the PubMed, Embase, Web of Science electronic databases to identify the literature using terms related to infection and AS. A search strategy combining "ankylosing spondylitis", "infection", "case-control studies" and "cohort studies" will be adopted. Searches will be restricted to English or Chinese language.

**Data management and selection process**

Endnote X9 will be used to catalogue search results. These will be de-duplicated using the "find duplicate" feature. According to the inclusion and exclusion criteria, titles and abstracts will be firstly screened, then full text of studies that could not be explicitly excluded based on their titles or abstracts will be screened with clear reasons documented. And reference lists of all included studies will be manually searched for additional studies. Two authors will independently screen the title, abstract, and full text. Any disagreement will be resolved through adjudication or by a third reviewer.

**Data extraction process**

Data from selected references will be extracted using Microsoft Excel 2019. Data extraction and quality assessment will be performed by two independent authors. Any disagreement will be settled by a third author.

**Data items**

Data will be extracted on:

The first name of the first author, year of publication, location, types of infection, definition of infection, definition of AS, study design, age, sex, sample size, follow-up duration/study period (years), adjustment for potential confounding, and estimates of associations.

**Quality assessment**

The quality of case-control and cohort studies will assess by the Newcastle–Ottawa Scale (NOS). Included studies will be scored across three categories with a maximum score of nine: (1) selection of subjects, (2) comparability of study groups, (3) the assessment of outcome/exposure. When a study awarded 7 or more score, 4, 5 or 6 score, and less than 4 score, it will be considered to be high, moderate, and low quality, respectively.

**Data synthesis**

A meta-analysis will be performed on association between infection and the risk of AS according to different study design. We will use Cochran’s *Q* test and the *I*^2^ statistic to detect heterogeneity among studies. *I*^2^ describes the percentage of total variation due to heterogeneity among studies rather than chance. In the presence of high heterogeneity (*I*^2^>50%), the Dersimonian and Laird random effects model (REM) will be adopted as the pooling method; otherwise, the Mantel-Haensze fixed effects model (FEM) will be adopted as the pooling method. Subgroup analysis or meta regression will be conducted to explore the possible sources of between study heterogeneity.

**Sensitivity analyses**

Sensitivity analyses will be performed to validate the stability of outcome. If the point estimate in the omitted analysis of an individual study is outside the 95% CI of the combined analysis, the individual study will be suspected to excessively influence the pooled effect value.

**Assessment of publication biases**

To assess publication bias, funnel plots will be used to plot the study effect size against sample size. Publication bias will also be assessed by Begg’s test.

**References**

1. Sieper J, Poddubnyy D. Axial spondyloarthritis. Lancet (2017) 390:73-84. doi:10.1016/s0140-6736(16)31591-4

2. Ajmani S, Keshri A, Srivastava R, Aggarwal A, Lawrence A. Hearing loss in ankylosing spondylitis. Int J Rheum Dis (2019) 22:1202-8. doi:10.1111/1756-185x.13560

3. Dean LE, Jones GT, MacDonald AG, Downham C, Sturrock RD, Macfarlane GJ. Global prevalence of ankylosing spondylitis. Rheumatology (Oxford) (2014) 53:650-7. doi:10.1093/rheumatology/ket387

4. Schlosstein L, Terasaki PI, Bluestone R, Pearson CM. High association of an HL-A antigen, W27, with ankylosing spondylitis. N Engl J Med (1973) 288:704-6. doi:10.1056/nejm197304052881403

5. Mear JP, Schreiber KL, Münz C, Zhu X, Stevanović S, Rammensee HG, et al. Misfolding of HLA-B27 as a result of its B pocket suggests a novel mechanism for its role in susceptibility to spondyloarthropathies. J Immunol (1999) 163:6665-70.

6. Goodall JC, Wu C, Zhang Y, McNeill L, Ellis L, Saudek V, et al. Endoplasmic reticulum stress-induced transcription factor, CHOP, is crucial for dendritic cell IL-23 expression. Proc Natl Acad Sci U S A (2010) 107:17698-703. doi:10.1073/pnas.1011736107

7. Zhang L, Hu Y, Xu Y, Li P, Ma H, Li X, et al. The correlation between intestinal dysbiosis and the development of ankylosing spondylitis. Microb Pathog (2019) 132:188-92. doi:10.1016/j.micpath.2019.04.038

8. Smith JA. Update on ankylosing spondylitis: current concepts in pathogenesis. Curr Allergy Asthma Rep (2015) 15:489. doi:10.1007/s11882-014-0489-6

9. Nielsen PR, Kragstrup TW, Deleuran BW, Benros ME. Infections as risk factor for autoimmune diseases - A nationwide study. J Autoimmun (2016) 74:176-81. doi:10.1016/j.jaut.2016.05.013

# Supplementary Table 2. Search strategy.

| Step | Searching strategy | Number of articles |
| --- | --- | --- |
| ***PubMed*** | | |
| #1 | "spondylitis, ankylosing"[MeSH Terms] | 15,353 |
| #2 | "infections"[MeSH Terms] | 2,805,810 |
| #3 | spondylitis, ankylosing OR spondyloarthritis ankylopoietica OR ankylosing spondylarthritis OR ankylosing spondylarthritides OR spondylarthritides, ankylosing OR spondylarthritis, ankylosing OR ankylosing spondylitis OR spondylarthritis ankylopoietica OR bechterew disease OR bechterew's disease OR bechterews disease OR marie-struempell disease OR marie struempell disease OR rheumatoid spondylitis OR spondylitis, rheumatoid OR spondylitis ankylopoietica OR ankylosing spondyloarthritis OR ankylosing spondyloarthritides OR spondyloarthritides, ankylosing OR spondyloarthritis, ankylosing[Title/Abstract] | 16,606 |
| #4 | enteritis OR enteritidis OR esoenteritis OR Salmonella OR Shigella OR Yersinia OR Campulobacter OR Escherichina OR pneumonia OR pulmonitis OR Klebsiella pneumoniae OR chlamydia OR mycoplasma OR urogenital infections OR bedsonia trachomatis OR paradentitis OR parodontitis OR periodontitis OR peridentitis OR porphyromonas OR prevotella OR tonsillitis OR amygdalitis OR paristhmitis OR infection of the upper respiratory tract OR upper respiratory tract inflection OR appendicitis OR ecphyaditis OR epityphlitis OR gastritis OR Helicobactor Pylori OR virus OR inframicrobe OR infect OR infection OR inflammation OR influence/infestation [Title/Abstract] | 2,383,873 |
| #5 | case-control study OR retrospective study OR cohort study OR prospective study OR longitudinal study OR follow-up study[All Fields] | 3,324,597 |
| #6 | #1 AND #2 | 2,812 |
| #7 | #3 AND #4 | 2,601 |
| #8 | #6 OR #7 | 5,095 |
| #9 | #8 AND #5 | 1,225 |
| ***Embase*** | | |
| #1 | 'ankylosing spondylitis'/exp | 30,887 |
| #2 | 'infection'/exp | 3,975,387 |
| #3 | 'ankylating spondylitis':ab,ti OR 'ankylopoietic spondylarthritis':ab,ti OR 'ankylopoietic spondylitis':ab,ti OR 'ankylosing spine':ab,ti OR 'ankylosing spondilitis':ab,ti OR 'ankylosing spondylarthritis':ab,ti OR 'ankylosing spondylarthrosis':ab,ti OR 'ankylosis spondylitis':ab,ti OR 'ankylotic spondylitis':ab,ti OR 'bechterew disease':ab,ti OR 'bekhterev disease':ab,ti OR 'morbus bechterew':ab,ti OR 'spinal ankylosis':ab,ti OR 'spine ankylosis':ab,ti OR 'spondylarthritis ankylopoietica':ab,ti OR 'spondylarthritis ankylosans':ab,ti OR 'spondylarthrosis ankylopoietica':ab,ti OR 'spondylitis ankylopoetica':ab,ti OR 'spondylitis ankylopoietica':ab,ti OR 'spondylitis, ankylosing':ab,ti OR 'spondyloarthritis ankylopoietica':ab,ti OR 'vertebral ankylosis':ab,ti | 1,098 |
| #4 | 'accidental infection':ab,ti OR 'acute infection':ab,ti OR 'autoinfection':ab,ti OR 'bacterial infections and mycoses':ab,ti OR 'bacteroid infection':ab,ti OR 'chronic infection':ab,ti OR 'dormant infection':ab,ti OR 'focal infection':ab,ti OR 'inapparent infection':ab,ti OR 'infection mechanism':ab,ti OR 'infection route':ab,ti OR 'infection, focal':ab,ti OR 'infections':ab,ti OR 'infectious disease':ab,ti OR 'infectivity':ab,ti OR 'latent infection':ab,ti OR 'prosthesis-related infections':ab,ti OR 'route of infection':ab,ti OR 'silent infection':ab,ti OR 'simultaneous infection':ab,ti OR 'enteritis':ab,ti OR 'enteritidis':ab,ti OR 'esoenteritis':ab,ti OR 'salmonella':ab,ti OR 'shigella':ab,ti OR 'yersinia':ab,ti OR 'campulobacter':ab,ti OR 'escherichina':ab,ti OR 'pneumonia':ab,ti OR 'pulmonitis':ab,ti OR 'klebsiella pneumoniae':ab,ti OR 'chlamydia':ab,ti OR 'mycoplasma':ab,ti OR 'urogenital infections':ab,ti OR 'bedsonia trachomatis':ab,ti OR 'paradentitis':ab,ti OR 'parodontitis':ab,ti OR 'periodontitis':ab,ti OR 'peridentitis':ab,ti OR 'porphyromonas':ab,ti OR 'prevotella':ab,ti OR 'tonsillitis':ab,ti OR 'amygdalitis':ab,ti OR 'antiaditis':ab,ti OR 'paristhmitis':ab,ti OR 'infection of the upper respiratory tract':ab,ti OR 'upper respiratory tract inflection':ab,ti OR 'appendicitis':ab,ti OR 'ecphyaditis':ab,ti OR 'epityphlitis':ab,ti OR 'gastritis':ab,ti OR 'helicobactor pylori':ab,ti OR 'virus':ab,ti OR 'inframicrobe':ab,ti | 1,918,297 |
| #5 | 'case-control study' OR 'retrospective study' OR 'cohort study' OR 'prospective study' OR 'longitudinal study' OR 'follow-up study' | 2,411,690 |
| #6 | #1 AND #2 | 4,779 |
| #7 | #3 AND #4 | 35 |
| #8 | #6 OR #7 | 4,798 |
| #9 | #8 AND #5 | 1,138 |
| ***Web of Science*** | | |
| #1 | TS=(Spondylitis,Ankylosing OR Spondyloarthritis Ankylopoietica OR Ankylosing Spondylarthritis OR Ankylosing Spondylarthritides OR Spondylarthritides, Ankylosing OR Spondylarthritis, Ankylosing OR Ankylosing Spondylitis OR Spondylarthritis Ankylopoietica OR Bechterew Disease OR Bechterew‘s Disease OR Bechterews Disease OR Marie-Struempell Disease OR Marie Struempell Disease OR Rheumatoid Spondylitis OR Spondylitis, Rheumatoid OR Spondylitis Ankylopoietica OR Ankylosing Spondyloarthritis OR Ankylosing Spondyloarthritides OR Spondyloarthritides, Ankylosing OR Spondyloarthritis, Ankylosing) | 14,610 |
| #2 | TS=(enteritis OR enteritidis OR esoenteritis OR Salmonella OR Shigella OR Yersinia OR Campulobacter OR Escherichina OR pneumonia OR pulmonitis OR Klebsiella pneumoniae OR chlamydia OR mycoplasma OR urogenital infections OR bedsonia trachomatis OR paradentitis OR parodontitis OR periodontitis OR peridentitis OR porphyromonas OR prevotella OR tonsillitis OR amygdalitis OR antiaditis OR paristhmitis OR infection of the upper respiratory tract OR upper respiratory tract inflection OR appendicitis OR ecphyaditis OR epityphlitis OR gastritis OR Helicobactor Pylori OR virus OR inframicrobe OR infect OR infection OR inflammation OR influence/infestation) | 1,838,622 |
| #3 | TS=(case-control study OR retrospective study OR cohort study OR prospective study OR longitudinal study OR follow-up study) | 1,489,830 |
| #4 | (#1 AND #2 AND #3) | 2,221 |

# Supplementary Table 3. Characteristics of included case-control studies on infections and AS.

| **First Author,**  **year** | **Study location** | **Types of**  **infection** | **Definition of infection** | **Definition of AS** | **Stars** |
| --- | --- | --- | --- | --- | --- |
| Chao, 2019^(27)^ | Taiwan | Tonsillitis | ICD-9-CM code 463,474 | ICD-9-CM code 720, 720.00 | 9 |
| Abbood,  2018^(28)^ | United  Kingdom | Oral Ulcers | The self-reported data on mouth/teeth or dental problems | a: self-reported whether they had been told by a doctor that they have some sort of severe non-cancer illness or disability  b: ICD-10 clinical records | 5 |
| Lindström,  2016^(26)^ | Sweden | Childhood  hospital infection | ICD-8, ICD-9, ICD-10 codes | ICD-8 code 712.40; ICD-9 code 720A; ICD-10 code M45 | 8 |
| Keller,  2013^(22)^ | Taiwan | Chronic Periodontitis | ICD-9-CM code 523.4 | ICD-9-CM codes 720 or 720.0 | 9 |
| Feng, 2011^(25)^ | China | *Chlamydia Pneumoniae* | ELISA | The modified AS New York criteria | 5 |
| Stone, 2004^(18)^ | Canada | *S. typhimurium*  *Y. enterocolitica*  *C. trachomatis*  *K. pneumoniae* | ELISA | The modified AS New York criteria | 9 |

AS, ankylosing spondylitis; *C. trachomatis*, *Chlamydia trachomatis*; ELISA, enzyme-linked immunosorbent assay; ICD-8, international classification of disease, eighth revision; ICD-9, international classification of disease, ninth revision; ICD-9-CM, international classification of disease, ninth revision, clinical modification; ICD-10, international classification of disease, tenth revision; *K. pneumoniae*, *Klebsiella pneumoniae*; *S. typhimurium*, *Salmonella typhimurium*; *Y. enterocolitica*, *Yersinia enterocolitica*. a represents case-control study of self-reported ankylosing spondylitis; b represents case-control study of clinical recorded ankylosing spondylitis.

# Supplementary Table 4. Other characteristics of included case-control studies on infections and AS.

| **First Author,**  **year** | **Sex (M/F)** | **Age [mean (SD) or median (range)]** | **Sample size** | **OR (95% CI)** | **Statistic used to**  **estimate OR** | **Adjustment** |
| --- | --- | --- | --- | --- | --- | --- |
| Chao, 2019^(27)^ | 163,905/95,109 | Case:42.6 (17.1)  Control:42.6 (17.1) | Case: 37,002  Control: 222,012 | 1.46 (1.43-1.50) | Conditional logistic regression analysis | Age, Comorbidity, History of periodontitis, appendicitis, Gender, |
| Abbood, 2018^(28)^ | a:  225,866/266,944  b:  224,930/265,997 | a: Case:58 (7.5)  Control:57 (8.1)  b: Case:57 (7.6)  Control:57 (8.1) | a: Case: 1,307  Control: 491,530  b: Case: 153  Control: 490,351 | a: 1.57 (1.31-1.88）  b: 2.17 (1.33-3.53) | Logistic regression model | Age, Gender, Smoking status, Body mass index, Alcohol consumption, Educational qualification level, |
| Lindström,  2016^(26)^ | 8,156/4,554 | Case:30 (6.2)  Control:30 (6.2) | Case: 2,453  Control: 10,257 | 1.08 (0.96-1.22)  Appendicitis:0.59 (0.41-0.83)  Respiratory tract infections:  1.24 (1.07-1.44)  Tonsillitis:1.31 (1.03-1.67) | Conditional logistic regression analysis | Sex, Year of birth, Age at first Spa-diagnosis, Pharmacological Treatment, AS-related inflammatory diseases |
| Keller, 2013^(22)^ | 23,892/17,034 | 27.2 (19.4) | Case: 6,821  Control: 34,105 | 1.84 (1.74-1.98) | Conditional logistic regression analysis | Sex, Age, Urbanization, Geographic Region, Level, Monthly income |
| Feng, 2011^(25)^ | 127/25 | Case:30.5 (7.9)  Control:29.1 (7.1) | Case: 79  Control: 73 | 0.70 (0.24-2.06) | Fisher's exact or the χ^2^ | NR |
| Stone, 2004^(18)^ | 54/42 | Case:39.4 (14.65)  Control:49.6 (20.5) | Case: 57  Control: 39 | *S.typhimurium*:1.30 (0.40-5.30)  *Y. enterocolitica*:  3.00 (0.60-30.50)  *C. trachomatis*:  3.60 (0.40-177.30)  *K. pneumoniae*:  0.40 (0.10-1.10) | Multiple logistic regression | Age, Sex, HLA-B27 status |

AS, ankylosing spondylitis; CI, confidence interval; *C. trachomatis*, *Chlamydia trachomatis*; *K. pneumoniae*, *Klebsiella pneumoniae*; M, men; NR, not reported; OR, odds ratio; SD, standard deviation; *S. typhimurium*, *Salmonella typhimurium*; W, women; *Y. enterocolitica*, *Yersinia enterocolitica*. a represents case-control study of self-reported ankylosing spondylitis; b represents case-control study of clinical recorded ankylosing spondylitis.

# Supplementary Table 5. Characteristics of included cohort studies on infections and AS.

| **First Author,**  **year** | **Study location** | **Types of infection** | **Definition of infection** | **Definition of AS** | **Follow-up**  **duration (years)** | **Stars** |
| --- | --- | --- | --- | --- | --- | --- |
| Damba, 2020^(20)^ | Canada | HIV | ICD-9 042–044 and Québec-specific ICD-9 7958 | ICD-9 code 720, ICD-10 code M45 | Mean (SD):  exposure:6.4(0.08)  control:4.9(0.03)  Median (IQR):  exposure:4.6(2.1-10.5)  control:3.6(1.3-7.6) | 9 |
| Wei, 2020^(19)^ | Taiwan | HPV | ICD-9-CM code 079.4, 078.1, 078.10-078.12, 078.19, 759.05, 795.09, 795.15, 795.19, 796.75, 796.79 | ICD-9-CM code 720.0 | 11^*^ | 9 |
| Bartels, 2020^(10)^ | Denmark | *Helicobacter Pylori* | UBT results | ICD-10 code M45.9 | Median:8 | 8 |
| Wei, 2020^(11)^ | Taiwan | *Candida albicans* | ICD-9-CM code 112 | ICD-9-CM code 720.0 | Median: exposure:7.42  control:7.33 | 9 |
| Chu, 2019^(24)^ | Taiwan | *Mycoplasma Pneumoniae* | ICD-9-CM codes 483.0 | ICD-9-CM code 720.0 | Mean: exposure:5.19  control:5.21 | 9 |
| Yen, 2017^(23)^ | Taiwan | HIV | ICD-9-CM code 042-044, 795.8 or V08 | ICD-9-CM code | 13^*^ | 7 |
| Nielsen, 2016^(17)^ | Denmark | Hospital infection | ICD-8, ICD-10 codes | ICD-8 code 712.49; ICD-10 code M45.9 | 34^*^ | 7 |

AS, ankylosing spondylitis; HIV, Immune thrombocytopenic purpura; HPV, Human Papillomavirus; ICD-8, international classification of disease, eighth revision; ICD-9, international classification of disease, ninth revision; ICD-9-CM, international classification of disease, ninth revision, clinical modification; ICD-10, international classification of disease, tenth revision; IQR, inter Quartile Range; SD, standard deviation; UBT, urea breath test.

^*^It is study period.

# Supplementary Table 6. Other characteristics of included cohort studies on infections and AS.

| **First Author,**  **year** | **Sex (M/F)** | **Age [mean (SD) or median (range)]** | **Sample size** | **No. of cases** | **RR/HR**  **(95% CI)** | **Statistic used to**  **estimate RR/HR** | **Adjustment** |
| --- | --- | --- | --- | --- | --- | --- | --- |
| Damba,2020^(20)^ | 15,761/4,977 | Exposure:  40.6 (0.17)  Control:  41.6 (0.09) | Exposure: 4,245  Control: 16,493 | Exposure: 21  Control: 33 | RR:1.82 (1.03-3.21) | Cox proportional hazard model | Age, sex, cohort entry data, Comorbidities |
| Wei,  2020^(19)^ | 159,595/171,975 | Birth cohort | Exposure: 66,314  Control: 265,256 | 836  Exposure:221  Control: 615 | HR:1.35 (1.15-1.58) | Multivariable Cox proportional hazards regression model | Age, Sex, Income,  Urbanization,  Comorbidities |
| Bartels,  2020^(10)^ | 33,627/22,367 | Exposure: 45.2  Control: 41.7 | Exposure: 10,975  Control: 45,019 | 59  Exposure:4  Control:55 | HR:0.23 (0.06-0.93) | Cox regression | Comorbidity, Age  Time of URT test,  Ethnicity, Alcohol  Gender, Obesity, Sex, |
| Wei,  2020^(11)^ | 12,727/110,373 | Birth cohort | Exposure: 61,550  Control: 61,550 | 449  Exposure:242  Control :207 | HR:1.19 (0.99-1.44) | Cox proportional hazard models | Income level,  Urbanization level,  Prior length of  hospital stay |
| Chu,  2019^(24)^ | 278,630/301,790 | Exposure:12 (18)  Control:12 (18) | Exposure: 116,084  Control: 464,336 | 27  Exposure:9  Control:18 | HR:2.45 (1.02-5.90) | Cox proportional hazard regression models | Sex, Comorbidities,  Age |
| Yen,  2017^(23)^ | Exposure：  18,727/1,727  Control: NR | 30.1 (11.0) | Exposure: 6,702  Control:2 million | Exposure:11  Control: NR | RR:0.86(0.43-1.55) | NR | NR |
| Nielsen,  2016^(17)^ | NR | NR | 4,500,000 | 4,096  Expose:1,342  Control:2,754 | RR:1.56 (1.46-1.68)  Bacterial:1.66 (1.51-1.82)  Viral:1.54 (1.33-1.78)  Other:1.56 (1.42-1.71) | Poisson regression | Calendar year, Sex, Age, Comorbidities |

AS, ankylosing spondylitis; CI, confidence interval; HR, hazard ratio; M, men; NR, not reported; RR, relative risk; SD, standard deviation; UBT, urea breath test; W, women.

# Supplementary Table 7. The subgroup analyses of the association between infections and the risk of AS based on possible factors.

| Subgroups | No. of  items | OR/RR/HR  (95% CI) | *I*^2^ (%) | Chi-square test  *P*-value |
| --- | --- | --- | --- | --- |
| **Case-control studies** |  |  |  |  |
| **Publication Year** |  |  |  | 0.655 |
| ≥2016 | 4 | 1.42 (1.16–1.74) | 88.8 |  |
| <2016 | 6 | 1.22 (0.66–2.29) | 49.9 |  |
| **Study location** |  |  |  | 0.730 |
| Asia | 3 | 1.58 (1.26–1.98) | 95.6 |  |
| Europe | 3 | 1.46 (1.03–2.08) | 88.0 |  |
| North America | 4 | 1.08 (0.40–2.89) | 29.8 |  |
| **Sample size** |  |  |  | 0.139 |
| ≥1000 | 5 | 1.51 (1.27–1.80) | 94.6 |  |
| <1000 | 5 | 0.89 (0.45–1.76) | 11.1 |  |
| **Definition of infection** |  |  |  | 0.201 |
| Clinical diagnosis | 3 | 1.44 (1.16–1.77) | 97.2 |  |
| Self-reporting | 2 | 1.70 (1.29–2.23) | 32.7 |  |
| Basic medical experiment | 5 | 0.89 (0.45–1.76) | 11.1 |  |
| **Cohort studies** |  |  |  |  |
| **Publication Year** |  |  |  | 0.595 |
| ≥2020 | 4 | 1.26 (0.97–1.64) | 64.7 |  |
| <2020 | 3 | 1.45 (0.94–2.22) | 53.7 |  |
| **Study location** |  |  |  | 0.938 |
| Asia | 4 | 1.27 (1.06–1.52) | 34.6 |  |
| Other^*^ | 3 | 1.24 (0.66–2.33) | 74.3 |  |
| **Sample size** |  |  |  | 0.529 |
| ≥100 thousand | 5 | 1.37 (1.15–1.62) | 68.7 |  |
| <100 thousand | 2 | 0.71 (0.09–5.37) | 86.6 |  |
| **Definition of infection** |  |  |  | 0.010 |
| Clinical diagnosis | 6 | 1.39 (1.19–1.63) | 62.3 |  |
| Basic medical experiment | 1 | 0.23 (0.06–0.91) | 0.0 |  |
| **Duration of follow-up** |  |  |  | 0.524 |
| ≥8 years | 4 | 1.29 (1.00–1.66) | 77.0 |  |
| <8 years | 3 | 1.51 (1.00–2.28) | 51.9 |  |

AS, ankylosing spondylitis; CI, confidence interval; HR, hazard ratio; OR, odds ratio, RR, relative risk.

^*^Other locations of studies include the Europe and the North America.
